# Estimating of Net Transition Probabilities in Triple Stages of Cigarette Consumption in Iranian Men

**Published:** 2018-10

**Authors:** Mahshid Aryanpur, Ahmad Khosravi, Mahmoud Yousefifard, Mostafa Hosseini, Alireza Oraii, Gholamreza Heydari, Mehdi Kazempour-Dizaji, Hooman Sharifi, Zahra Hessami, Hamidreza Jamaati

**Affiliations:** 1 Tobacco Prevention and Control Research Center, National Research Institute of Tuberculosis and Lung Diseases (NRITLD), Shahid Beheshti University of Medical Sciences, Tehran, Iran,; 2 Department of Epidemiology, School of Public Health, Shahroud University of Medical Sciences, Shahroud, Iran,; 3 Physiology Research Center, Faculty of Medicine, Iran University of Medical Sciences, Tehran, Iran,; 4 Department of Epidemiology and Biostatistics, School of Public Health, Tehran University of Medical Sciences, Tehran, Iran,; 5 Department of Medicine, Tehran University of Medical Sciences, Tehran, Iran,; 6 Mycobacteriology Research Center, Biostatistics Unit, NRITLD, Shahid Beheshti University of Medical Sciences, Tehran, Iran.

**Keywords:** Smoking, Tobacco, Tobacco Use, Epidemiology, Prevalence

## Abstract

**Background:**

The present study was designed to estimate the net transition probabilities in triple stages of cigarette consumption in Iranian men over 15 years old.

**Materials and Methods:**

Data from the national surveillance of risk factors of non-communicable diseases in 2011 were entered in the present study. Data of 3130 Iranian men between the ages of 15 and 69 years old were included. Individuals were divided to three groups of current smoker, past smoker and nonsmoker based on cigarette consumption. Afterwards, net transition probability of different stages of cigarette consumption over a year was assessed.

**Results:**

Net transition probability from nonsmoker to smoker was at its highest level until 30 years of age at 19.1 per 1000 men and then net transition reduces to reach zero per 1000 men at the age of 45 years old. However, net transition probability from smoker to nonsmoker was at a very low level until 45 years of age but, it increases afterwards to reach a plateau at the age of 64 years old. Net transition probability from smoker to nonsmoker is estimated to be 23.1 per 1000 men at the age of 69 years old.

**Conclusion:**

For the first time, the present study has estimated the transition probabilities in different stages of cigarette consumption in Iranian adults. Findings showed that risk of becoming a smoker in younger individuals is much higher than the risk in middle-aged and old population. However, tendency to quit smoking is increased after the age of 45 years old. Therefore, health policy makers should concentrate on younger age groups in their preventive strategies regarding control of tobacco consumption.

## INTRODUCTION

Assessing the transition of individuals between different groups of cigarette consumption in a time period is defined as transition probability which has a great importance in health policy making systems (1). With the help of the mentioned concept, it can be predicted that how many nonsmokers will become smoker and how many will quit smoking in the following year or longer time periods. Future cigarette consumption status of the country can be acknowledged based on mentioned estimations and such measures will help health policy makers in application of preventive strategies (2, 3).

Cigarette smoking is considered the most important preventable risk factor of death across the world. Cigarette smoking is the risk factor of many diseases such as cancer, cardiovascular diseases, pulmonary fibrosis, and many other non-communicable diseases (4–7). Relative risk of getting a cancer is 23 times greater in smokers compared to nonsmokers (4,6). Cigarette smoking is responsible for the death of more than half of its long-term consumers as almost 5 million adults lost their lives due to cigarette smoking in 2000 (about 12% of all deaths in 2000) and this amount is estimated to reach 8.3 million individuals in 2030. Seventy percent of these deaths occurred in developing countries (4). World Health Organization (WHO) estimates that there are 1.3 billion smokers across the world consisting one third of population over 15 years old which will ultimately reach 2 billion people if the mentioned pattern of cigarette smoking remains until 2030 (8). Cigarette smoking prevalence in Iran is reported to be about 19% in men and 1.5% in women with a total prevalence of 11.9% in Tehran and 12.5% across the country (9, 10).

Cigarette consumption is divided into three stages in an epidemiological view including current smokers, past smokers and nonsmokers. Individuals who have smoked at least 100 cigarettes either daily or occasionally are considered current smokers. Individuals who were used to smoke in a daily manner and have quitted are considered past smoker and individuals who have not smoked a cigarette are considered nonsmokers (1).

Although there are many studies across the world assessing cigarette smoking and its different consumption stages (1, 11–15), there are no studies assessing net transition probability in different stages of cigarette consumption in Iranian adults. Hence, the goal of the present study was to estimate the net transition probabilities in triple stages of cigarette consumption in Iranian men using data from the national surveillance of risk factors of non-communicable diseases in 2011.

## MATERIALS AND METHODS

### Study setting and participants

Data from the national surveillance of risk factors of non-communicable diseases in 2011 were entered (3130 men between the ages of 15 and 69 years old). In the national surveillance of risk factors of non-communicable diseases in 2011, data of 7510 people were gathered in a systematic approach and multi-stage cluster sampling using databases of geography department and postal codes of Iran’s post company. Distribution of selected clusters was proportionate to the number of households in different postal areas of the state and also population ratio of cities and villages. Minimum sample size was 200 individuals in the least populated state of the country and maximum sample size was 800 individuals in state samples. Data of cigarette consumption status of 3130 men between the ages of 15 and 69 years old was recorded. In the mentioned sampling, four age groups of 25 to 34, 35 to 44, 45–54 and 55 to 64 years old were selected including equal number of men and women. Technical committee of world health organization suggests at least 1000 individuals for the mentioned age and sex based groupings. Hence, sample size in mentioned groups is more than 1000 in the present study.

### Variables

Data cleaning was performed before analysis of gathered data. Data cleaning needs to be highly accurate and its correct application leads us to more reliable results. Hence, the accuracy of entering data and amount of missing were assessed. The way of entering data was the first source of error. The mentioned error which is a common problem in large databases was assessed. Therefore, frequency of data of each variable was obtained first and then minimum and maximum amounts of each column were assessed. This method gave us the opportunity to get a quick look at the data. In addition, both very high and very low amounts which could not be attributed to cigarette smoking were evaluated. At the end, the amount of missing data was specified. The amount of observed missing was less than 0.6% which could be ignored. Statistical analyses were done when accuracy of data was assured.

Cigarette consumption status (and its kinds) was assessed in Iranian men between 15 and 69 years old. Variables of age, age at which first cigarette was smoked and age at which cigarette smoking was quitted were measured. Prevalence of different cigarette consumption stages in the population was assessed.

Individuals over 15 years old were categorized into 3 stages based on cigarette consumption. Individuals who have smoked either daily or occasionally are considered current smokers. Individuals who were used to smoke in a daily manner and have quitted in the present day are considered past smoker and individuals who have not smoked a cigarette (including its different kinds such as factory cigarettes, hand-made cigarettes and cigars) are considered nonsmokers.

### Outcome

Net transition probability in different stages of cigarette consumption was the most important assessed outcome of the present study. Net transition describes the differences between inflow and outflow from one state to another state. In the present study, movement of individuals between different stages of cigarette consumption within a year was defined as transition.

### Statistical analyses

Data analysis was performed using R software. Estimation of net transition probabilities in three stages of nonsmoker, current smoker and past smoker was performed in two steps. In the first step, specific age prevalence of three stages of nonsmoker, current smoker and past smoker were smoothed and in the second step, net transition probabilities in different stages was estimated with the help of methods used for solving transportation problems. Methodology of estimating the net transition probabilities are fully explained in the study by Kassteele et al. (16). Briefly in the first step, age specific prevalence of triple stages of cigarette consumption in study population was smoothed using a multinomial p-spline method. Noise reduction in probabilities in the triple stages of cigarette consumption was the goal of the mentioned step. In the second step, transition probability was estimated by the help of idea used in solving transportation problems. Estimations are minimized using methods used in operation research models in order to increase the power of estimated transitions. In the mentioned method, smoothed prevalence of different stages in two consecutive years was used to estimate net transition probability. These estimations were done based on the two following assumptions:

A) Net Transition probabilities between triple stages are stable between two consecutive ages over time. We assumed that sudden jumps between consecutive ages in the terms of transitional probabilities are unlikely, B) Changes among the smoothed prevalence of triple stages of cigarette consumption between two consecutive ages are similar to transition probabilities among the triple stages of cigarette consumption after a year increase in age. It means that one year transitional probability for 15 years old subjects is similar to differences between smoothed prevalence of smoking stages of 15 and 16 years old subjects.

After accepting mentioned assumptions, number of individuals in two consecutive ages can be used for estimations. As number of individuals in two consecutive ages is not always equal in a cross-sectional study, we used change as prevalence share of each stage in two consecutive ages. Hence, sum of prevalence shares of all stages equals to one. Kassteele and colleagues (16) address that actual prevalence of stages of cigarette consumption is a smoothed function based on age.

Net transition (***τ_ij_***) is positive and above zero for defined stages as retrograde net transition does not exist (meaning that transition from smoker to nonsmoker does not exist). Net transition from stage i at the age of a-1 to stage j at the age of a (***τ_ij_*** ≥ **0**). Hence, net transition of τ′_ij_ equals:

$$\tau_{ij}^{\prime }\{\begin{array}{ll} \tau_{ij}-\tau_{ji} & \tau_{ij}>\tau_{ji}\\ 0 & \mathrm{otherwise} \end{array}$$

In the second step with the help of transportation problem, net transition was defined as decision variable, smoothed prevalence of age a-1 as supply, smoothed prevalence of age as demand and transition matrix (C _ij _) as shipping cost from one stage to the next stage in a function and the mentioned function is minimized in all ages. In the present study, shipping cost matrix is defined based on the following: A) Staying in the same stage after a year is defined as the cheapest situation, B) In case of transition, one stage of transition is cheaper than two stages of transition and advancing two steps forward in stages costs. In other words, when a nonsmoker becomes a smoker it costs less than when a nonsmoker becomes a smoker after a year and then transits to being a past smoker (transition has occurred in two steps in the latter).

$$\min\;j=\sum_{i=1}^{k}\sum_{j=1}^{k}c_{ij}\tau_{ij}^{\prime }$$

## RESULTS

Data of 3130 men over 15 years old were entered. Mean age was 40.6±0.25 years old. Mean age of the beginning of cigarette smoking was 24.0 years old (95%CI: 22.9–25.0). Cigarette consumption had a prevalence of 21.1% among men (95%CI: 19.9–22.4) and prevalence of individuals who had quitted smoking was 7.1% (95%CI: 6.3–7.9). The highest prevalence of cigarette consumption was observed in the age group of 35 to 64 years old. Table 1 shows different stages of cigarette consumption based on age groups.

**Table 1.** Observed prevalence of smoking stages in population

**Age group**	**Nonsmoker (95% CI)**	**Smoker (95% CI)**	**Past smoker (95% CI)**
**15–24**	91.2 (89.3–92.8)	6.3 (4.9–7.9)	2.5 (1.7–3.7)
**25–34**	74.9 (72.1–77.6)	21.4 (18.9–24.1)	3.4 (2.7–5.1)
**35–44**	59.6 (55.6–63.4)	33.7 (30.0–37.6)	6.7 (5.0–9.0)
**45–54**	60.3 (56.0–64.5)	28.8 (25.0–32.9)	10.8 (8.4–13.9)
**55–64**	63.0 (60.0–65.9)	25.1 (22.5–27.9)	11.9 (10.0–14.0)
**65–70**	68.2 (63.3–73.7)	19.6 (15.5–24.6)	11.7 (8.5–16.0)
**Total**	71.8 (70.4–73.1)	21.1 (19.9–22.4)	7.1 (6.3–7.9)

Data are presented as percentage with 96% confidence interval (95% CI).

**Figure 1.**
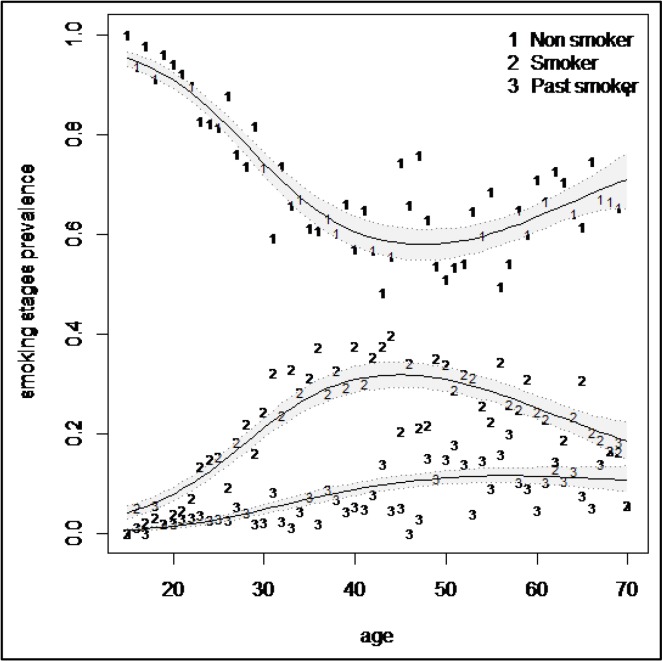
Trend of smoothed prevalence of smoking stages in studied population

Smoothed prevalence of triple stages of cigarette consumption is depicted in figure 1 for age specific prevalence in Iranian men. As shown, prevalence of cigarette smoking has had an increasing trend until the age of 45 years old and then the prevalence of cigarette consumption has slowly decrease after the age of 45 years old. Number of past smokers has had a slowly increasing trend until the age of 50 years old and then has reached a plateau.

**Figure 2.**
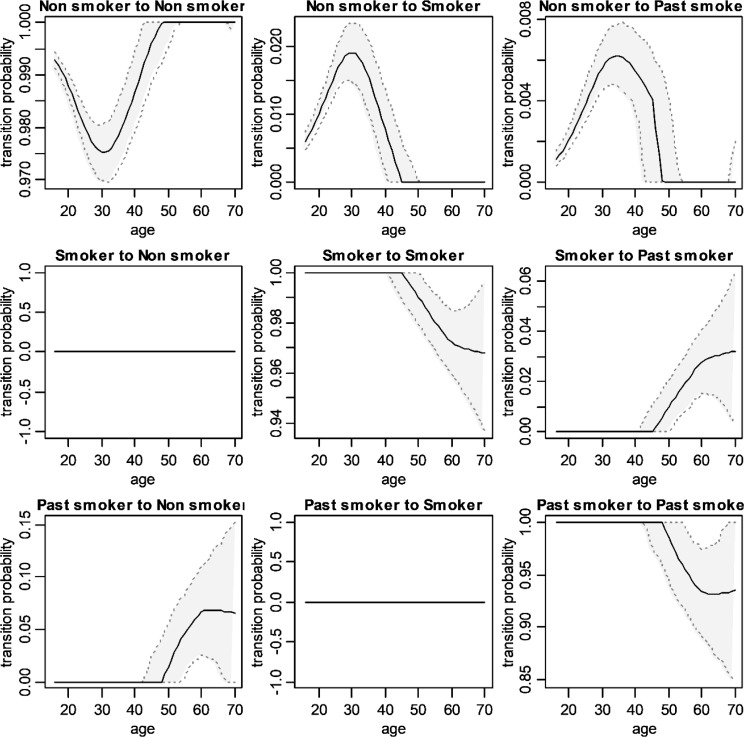
Net Transition probability among smoking stages in Iranian men.

Estimation of net transition in different stages of cigarette consumption is shown in figure 2 and table 2. As shown, net transition probability from nonsmoker to smoker reaches its maximum at the age of 30 years old (19.1 individuals per 1000 people) and then this net transition decreases to reach zero at the age of 45 years old. In other words, the probability of a nonsmoker becoming a smoker after the age of 45 years old is extremely low. In addition, net transition probability from current smoker to nonsmoker is very low until the age of 45 years old but, the mentioned probability increases to reach a plateau at the age of 64 years old. Transition probability from current smoker to nonsmoker was estimated to be 33.1 individuals per 1000 people at the age of 69 years old (Figure 2 and Table 2).In addition, analysis showed that some nonsmokers start smoking and then quit smoking until the age of 50 years old. The mentioned probability reaches its maximum at the ages of 30 to 35 years old (5.9 to 6.2 individuals per 1000 people) and becomes almost zero after the age of 50 years old (Figure 2 and Table 2).

**Table 2.** Age adjusted smoothed prevalence (%) of smoking stages in population

**Age**	**Nonsmoker**	**smoker**	**Past smoker**	**Net Transition probability (per 1000 person)**		

				**Nonsmoker to smoker**	**Smoker to past smoker**	**nonsmoker to past smoker**
15	95.2	4.1	0.66	6.0	0	1.1
16	94.5	4.7	0.76	6.9	0	1.3
17	93.8	5.3	0.89	7.8	0	1.6
18	92.9	6.1	0.10	8.9	0	1.8
19	91.9	6.9	1.2	10.0	0	2.1
20	90.8	7.8	1.4	11.2	0	2.4
21	89.5	8.8	1.6	12.5	0	2.7
22	88.2	9.9	1.8	13.7	0	3.1
23	86.7	11.2	2.1	14.9	0	3.5
24	85.1	12.5	2.4	16.1	0	3.9
25	83.4	13.8	2.8	17.1	0	4.3
26	81.6	15.2	3.1	17.9	0	4.7
27	79.8	16.7	3.5	18.6	0	5.0
28	77.9	18.2	3.9	19.0	0	5.4
29	76.0	19.7	4.3	19.1	0	5.6
30	74.1	21.1	4.7	19.0	0	5.9
31	72.3	22.5	5.2	18.5	0	6.1
32	70.5	23.9	5.6	17.8	0	6.2
33	68.8	25.1	6.1	16.7	0	6.2
34	67.2	26.3	6.5	15.5	0	6.2
35	65.8	27.3	6.9	14.1	0	6.1
36	64.4	28.4	7.3	12.6	0	6.0
37	63.2	29.1	7.7	11.0	0	5.9
38	62.2	29.8	8.1	9.4	0	5.7
39	61.2	30.3	8.4	7.8	0	5.5
40	60.4	30.8	8.8	6.2	0.01	5.2
41	59.7	31.2	9.1	4.7	0.05	4.9
42	59.2	31.5	9.4	3.3	0.2	4.5
43	58.7	31.6	9.6	2.1	0.5	3.9
44	58.3	31.7	9.9	1.3	1.0	3.2
45	58.1	31.7	10.1	0.7	2.0	2.5
46	57.9	31.7	10.4	0.4	3.4	1.9
47	57.9	31.5	10.6	0.2	4.7	1.4
48	57.8	31.4	10.8	0.1	6.2	0.9
49	57.9	31.1	10.9	0.03	8.0	0.5
50	58.1	30.8	11.1	0.01	9.9	0.25
51	58.3	30.4	11.2	0.003	11.8	0.1
52	58.6	30.0	11.3	0.002	13.9	0.05
53	59.0	29.5	11.4	0.0017	16.0	0.03
54	59.5	29.0	11.5	0	17.9	0.02
55	60.0	28.4	11.5	0	19.7	0.01
56	60.6	27.8	11.5	0	21.3	0
57	61.2	27.2	11.6	0	23.1	0
58	61.9	26.5	11.5	0	24.8	0
59	62.7	25.8	11.5	0	26.4	0
60	63.4	25.1	11.4	0	27.7	0
61	64.2	24.4	11.4	0	28.7	0
62	65.0	23.7	11.3	0	29.3	0
63	65.8	23.0	11.2	0	29.9	0
64	66.6	22.3	11.2	0	30.4	0
65	67.3	21.6	11.1	0	30.8	0
66	68.1	20.9	11.0	0	31.3	0
67	68.8	20.3	10.9	0	31.7	0
68	69.5	19.6	10.8	0	32.2	0
69	70.3	19.0	10.7	0	32.7	0
70	70.9	18.4	10.6	0	33.1	0

## DISCUSSION

The present study showed that net transition probability from nonsmoker to smoker reaches its maximum at the age of 30 years old and reduces afterwards to reach zero at the age of 45 years old. Net transition probability from current smoker to nonsmoker is very low until the age of 45 years old but, the mentioned probability increases to reach a plateau at the age of 64 years old. These findings indicate that most changes in tendency to cigarette smoking occur before the age of 45 years old. Hence, health policy makers should concentrate on younger age groups in their preventive strategies regarding control of cigarette consumption.

For the first time, the present study has assessed the net transition in different stages of cigarette consumption in Iranian adults. Hence, direct comparison of results of the present study with other similar studies is not possible. Only in one study by Khosravi et al. with the goal of estimating transition probability in different stages of cigarette consumption in Iranian adolescents, it was shown that the probability of nonsmoker boys becoming smoker is 8.9-fold greater compared to same aged girls (1). Similar to the present study, Fotouhi et al. showed that prevalence of cigarette consumption is significantly lower in ages above 55 years old (9). This finding is concordant with other studies reporting that tobacco consumption usually increases until 5th and 6th decade of life (17–20) and then decreases (6,19). On the other hand, prevalence of individuals quitting cigarette smoking increases in higher ages (17,19).

Probability of staying in a stage of cigarette consumption until the next time period is considered probability of staying and if transition to another stage happens it is considered probability of transition. Estimation of these transition probabilities is done using longitudinal studies assessing cigarette consumption status in different stages in a repetitive manner. However, less longitudinal studies are conducted due to their time consuming nature, high cost, problems regarding follow ups and accuracy of answers regarding cigarette consumption (1). Therefore, statistical methodology used in the present study has created an artificial longitudinal cohort based on data of a cross-sectional study in order to overcome limitations of a longitudinal study (16).

## CONCLUSION

For the first time, the present study has estimated the transition probability in different stages of cigarette consumption in Iranian adults. The present study which has used data of the national surveillance of risk factors of noncommunicable diseases showed that probability of becoming a smoker reaches its maximum at the age of 30 years old and then net transition decreases to reach zero at almost the age of 45 years old. In addition, tendency to quit smoking is increased after the age of 45 years old compared to younger ages. Therefore, health policy makers should concentrate on younger age groups in their preventive strategies regarding control of tobacco consumption.
